# Surgical Management of Ischemic Heart Disease Patients With Left Ventricular Dysfunction in Lower-Middle-Income Countries: Our Strategies and Experience at the Medical Teaching Institute-Hayatabad Medical Complex (MTI-HMC) Peshawar, Pakistan

**DOI:** 10.7759/cureus.77063

**Published:** 2025-01-07

**Authors:** Muhammad Aasim, Raheel Khan, Atta ul Mohsin, Jibran Ikram, Raheela Aziz, Ayesha Zahid

**Affiliations:** 1 Cardiac Surgery, Hayatabad Medical Complex Peshawar, Peshawar, PAK; 2 Cardiovascular Medicine, Hayatabad Medical Complex Peshawar, Peshawar, PAK

**Keywords:** coronary artery bypass grafting, impaired left ventricular function, low ejection fraction

## Abstract

Introduction and objectives

Severe left ventricular dysfunction (LVD) in coronary artery disease (CAD) is linked to high risks and limited outcomes. Coronary artery bypass grafting (CABG) remains a key surgical intervention for these patients. This study aimed to assess hospital and short-term outcomes in patients with severe LVD undergoing isolated CABG and identify predictors of adverse outcomes.

Methodology

We conducted a retrospective study of 454 patients who underwent CABG for CAD with significant LVD at Hayatabad Medical Complex between 2018 and 2024. Data were extracted from clinical records and analyzed statistically to evaluate outcomes and predictors.

Results

The study included 454 patients with a mean age of 58.14 ± 9.576 years and a mean ejection fraction of 35.59 ± 3.996%. There were 396 (87.2%) male patients with common comorbidities, including hypertension (122, 26.9%), diabetes mellitus (88, 19.4%), and smoking (39, 8.6%). Intraoperative findings showed a mean cardiopulmonary bypass (CPB) time of 155.94 ± 38.120 minutes, with 451 (99.3%) achieving LIMA (left internal mammary artery) to LAD (left anterior descending artery) revascularization. Postoperative in-hospital mortality was 21 (4.6%), re-intubation occurred in 18 (4.0%), and arrhythmias were observed in 26 (5.7%). Wound infections were minimal (444 (97.8%) without infection), and 406 (89.4%) underwent elective CABG, while nine (2.0%) had emergent CABG, mostly due to ventricular septal rupture.

Conclusion

CABG remains a vital surgical intervention for patients with severe LVD, offering favorable short-term outcomes despite the inherent risks. Key factors contributing to these results include comprehensive myocardial revascularization, effective use of internal mammary artery grafting, and advanced myocardial protection strategies. This study highlights the potential of CABG to improve survival and functional outcomes in this high-risk population.

## Introduction

Coronary artery disease (CAD) is the leading cause of heart disease worldwide and is the third most common cause of death in both men and women globally [1]. Coronary artery bypass grafting (CABG) remains the most frequently performed surgical intervention for individuals with multi-vessel CAD [2]. Numerous perioperative risk factors have been identified that influence outcomes following CABG. These include traditional predictors such as advanced age, female gender, diabetes mellitus, hypertension, chronic obstructive pulmonary disease (COPD), renal dysfunction, left main stem disease, and decreased left ventricular ejection fraction (LVEF) [3,4].

Preoperative left ventricular dysfunction (LVD) is a significant risk factor associated with higher early and late mortality after revascularization. Managing CABG in patients with LVD remains a challenging surgical task, requiring careful evaluation and planning to improve outcomes [5]. Revascularization in patients with severely impaired LV function raises several concerns, including poor prognosis, advanced CAD, and reduced graft durability. These issues are often due to inadequate distal blood flow in patients with severe LVD and extensive myocardial scarring. Consequently, the relief of angina after revascularization may be limited, leading to ongoing debate about the role and effectiveness of isolated CABG in this high-risk patient group [6]. Although surgical techniques, myocardial protection strategies, and postoperative care have advanced, the risk associated with surgery continues to remain high [7-9]. Several studies have demonstrated that patients experience improved quality of life and a higher ejection fraction following coronary surgery compared to those on ongoing medical therapy [10-13].

CABG in patients with severe myocardial dysfunction helps preserve viable myocardium, prevents further myocardial damage, and promotes the recovery of systolic function in hibernating or ischemic myocardial segments. However, the postoperative mortality rate in this patient population varies widely, ranging from 1.6% to 40% [14]. The aim of this study was to evaluate the operative outcomes in patients with severe LVD who underwent CABG in combination with additional procedures, including ventricular septal rupture (VSR) repair, mitral valve repair, mitral valve replacement, and aortic valve replacement. Furthermore, the study aimed to analyze how preoperative and surgical factors impacted early postoperative outcomes to identify key predictors of complications and risks.

## Materials and methods

Study design and setting

We conducted a retrospective cohort study of 454 consecutive patients eligible for CABG between 2018 and 2024 at Hayatabad Medical Complex in Peshawar, Pakistan. Ethical approval was obtained from the institutional research and ethics board.

Patient population

The mean age of the study population was 58.14 ± 9.576 years, and the mean LVEF was 35.59 ± 3.996. Patient demographics, including age, sex, and risk factors such as diabetes, hypertension, smoking, renal failure, cerebrovascular disease, and a history of prior percutaneous coronary intervention, were collected.

Data collection

Data were collected retrospectively from hospital records. The collected information included preoperative, intraoperative, and postoperative variables. All patients underwent preoperative two-dimensional echocardiography and coronary angiography.

Surgical procedure

All patients followed a standardized anesthetic protocol, which included fentanyl, midazolam, and pancuronium for induction, with maintenance provided using isoflurane and propofol. CABG was performed through a median sternotomy, with the left internal mammary artery (LIMA) and saphenous vein harvested for grafting. Cardiopulmonary bypass (CPB) was established after administering heparin (300-400 IU/kg) to ensure a target-activated clotting time of at least 400 seconds. Myocardial protection was achieved using a del Nido cardioplegia, administered through the aortic root and retrogradely via the coronary sinus. Complete revascularization was performed in all patients using conventional surgical techniques. After all anastomoses were completed and the patient was weaned off CPB, heparin was reversed with protamine to restore normal clotting function.

Postoperative management

At the conclusion of surgery, patients were transferred to the intensive care unit (ICU) for close monitoring and management. This included optimizing ventilatory support, ensuring hemodynamic stability, maintaining normothermia, and balancing fluid, electrolyte, and temperature status. Extubation was performed once patients regained consciousness, demonstrated sufficient respiratory effort and acceptable arterial blood gas levels, achieved hemodynamic stability, maintained normothermia, and showed no signs of excessive bleeding.

Outcome variables

The primary postoperative endpoint was all-cause mortality. Secondary outcomes included re-operation for bleeding or tamponade, arrhythmias, respiratory failure, stroke, ventilation duration, heart failure, re-intubation, length of ICU stay, in-hospital death, early mortality (≤30 days), and wound infections (monitored for one month postoperatively).

Statistical analysis

Data were collected retrospectively and analyzed using IBM SPSS Statistics for Windows, Version 20 (Released 2011; IBM Corp., Armonk, New York). Continuous variables were expressed as mean ± standard deviation, while categorical variables were reported as frequencies and percentages. Associations between preoperative, intraoperative, and postoperative variables and outcomes were assessed to identify predictors of complications and mortality.

## Results

The study included a total of 454 patients, with a mean age of 58.14 ± 9.576 years. The average ejection fraction among the participants was 35.59 ± 3.996%, reflecting a high prevalence of LVD within the study population. In terms of gender distribution, 396 (87.2%) of the patients were male, while females were 58 (12.8%). These demographic details provide insight into the baseline characteristics of patients undergoing CABG with severe LVD in this cohort (Table 1).

**Table 1:** Baseline demographic and clinical characteristics of the study population undergoing coronary artery bypass grafting (CABG)

Variable		Frequency/Mean (SD)	Percent (%)
Age (years)		58.14 ± 9.576	-
Ejection fraction (EF) %		35.59 ± 3.996	-
Gender	Male	396	87.2%
	Female	58	12.8%

Among the study participants, 122 (26.9%) had a history of hypertension, while 88 (19.4%) had diabetes mellitus. Smoking was present in 39 (8.6%) of the patients. Preoperative renal impairment was observed in 10 (2.2%) of the participants. Additionally, five (1.1%) patients had a prior history of cerebrovascular accident (CVA), and 31 (6.8%) had undergone prior percutaneous coronary intervention (PCI). These findings highlight the common presence of multiple cardiovascular risk factors and comorbidities in patients with severe LVD undergoing CABG (Table 2).

**Table 2:** Prevalence of comorbidities among patients undergoing coronary artery bypass grafting (CABG) CVA: cerebrovascular accident; PCI: percutaneous coronary intervention

Condition	Frequency	Percent (%)
Hypertension (HTN)	122	26.9%
Diabetes mellitus (DM)	88	19.4%
Smokers	39	8.6%
Pre-op renal impairment	10	2.2%
Prior CVA	5	1.1%
Prior PCI	31	6.8%

The mean duration of CPB was 155.94 ± 38.120 minutes, while the mean cross-clamp time was 93.64 ± 22.445 minutes. The average length of ICU stay following the procedure was 45.91 ± 12.262 hours, and the mean duration of ventilation was 10.43 ± 15.647 hours. Complete revascularization was achieved using LIMA to LAD (left anterior descending artery) in 451 (99.3%) of the patients. Re-exploration was required in 14 (3.1%) cases, while off-pump procedures were performed in 11 (2.4%). Cardioplegia was administered using different methods: 426 (93.8%) of patients received both antegrade and retrograde cardioplegia, 17 (3.7%) received antegrade cardioplegia alone, and 11 (2.4%) did not receive cardioplegia. These intraoperative findings underscore the surgical strategies and variability observed in the CABG procedures for patients with severe LVD (Table 3).

**Table 3:** Intraoperative variables in coronary artery bypass grafting (CABG) procedures LIMA: left internal mammary artery; LAD: left anterior descending artery; ICU: intensive care unit; CPB: cardiopulmonary bypass

Variable	Frequency/Mean (SD)	Percent (%)
CPB time (minutes)	155.94 ± 38.120	-
Cross-clamp time (minutes)	93.64 ± 22.445	-
ICU stay (hours)	45.91 ± 12.262	-
Ventilation time (hours)	10.43 ± 15.647	-
LIMA to LAD	451	99.3%
Re-exploration	14	3.1%
Off-pump procedures	11	2.4%
Cardioplegia methods: none	11	2.4%
Cardioplegia methods: antegrade	17	3.7%
Cardioplegia methods: both	426	93.8%

Postoperative complications were assessed to determine early surgical outcomes following CABG. Postoperative renal failure occurred in eight (1.8%) patients, while postoperative CVA was observed in three (0.7%). Additionally, postoperative respiratory failure was reported in nine (2.0%) cases. In-hospital mortality was observed in 21 (4.6%) of the cohort, and re-intubation was required in 18 (4.0%) patients. Arrhythmia was noted in 26 (5.7%) of the cases. These findings highlight the notable risks and complications associated with CABG in patients with severe LVD, emphasizing the need for careful monitoring and management in the postoperative period (Table 4).

**Table 4:** Postoperative outcomes CVA: cerebrovascular accident

Outcome	Frequency	Percent (%)
Post-op renal failure	8	1.8%
Post-op CVA	3	0.7%
Post-op respiratory failure	9	2.0%
In-hospital mortality	21	4.6%
Re-intubation	18	4.0%
Arrhythmia: yes	26	5.7%

Postoperative wound infections were evaluated as part of the surgical outcomes, and the findings demonstrated a low overall rate of complications. A total of 444 (97.8%) patients had no evidence of wound infection. However, superficial surgical site infection (SSWI) was identified in six (1.3%) cases, while deep surgical site infection (DSWI) and mediastinitis were each observed in two (0.4%) patients. Although these infection rates were relatively low, their presence highlights the importance of maintaining meticulous surgical technique, adhering to strict wound care protocols, and ensuring prompt identification and management of complications in the postoperative period (Table 5).

**Table 5:** Incidence and types of postoperative wound infections in coronary artery bypass grafting (CABG) patients

Type of Wound Infection	Frequency	Percent (%)
None	444	97.8%
Superficial surgical site infection (SSWI)	6	1.3%
Deep surgical site infection (DSWI)	2	0.4%
Mediastinitis	2	0.4%

The urgency of surgical intervention was categorized to assess the clinical presentation and associated risk factors in the study population. Among the 454 patients included, 406 (89.4%) underwent elective CABG, representing planned and stable revascularization procedures. Thirty-nine (8.6%) required urgent CABG due to worsening clinical conditions, while nine (2.0%) underwent emergent CABG, most associated with VSR, a life-threatening complication following acute myocardial infarction. These findings highlight the diverse clinical presentations and the varying levels of risk involved in CABG procedures, with emergent surgeries carrying the highest degree of complexity and hemodynamic instability (Table 6).

**Table 6:** Urgency of surgery

Category	Frequency	Percent (%)
Elective	406	89.4%
Urgent	39	8.6%
Emergent	9	2.0%

## Discussion

CAD is the leading underlying cause of heart failure, impacting an estimated 20 million individuals across Europe and the United States [15,16]. Atherosclerosis, LV systolic dysfunction, and heart failure are progressive conditions. Therefore, it is reasonable to assume that targeted medical and surgical treatments focused on these conditions can slow the progression of heart failure, lower morbidity, and extend life expectancy [17].

According to the latest clinical guidelines, CABG is recommended for patients with LVD caused by CAD who present with symptoms of angina and/or evidence of complex CAD [18]. Patients with severe CAD and significantly impaired LV function represent a high-risk population undergoing CABG. Even in recent studies, these patients have demonstrated high mortality rates, ranging from 2.7% to 33%, and high morbidity rates, ranging from 30% to 67%, highlighting the challenges and risks associated with CABG in this vulnerable group [19-21].

Heart transplantation is an effective treatment option for end-stage heart failure; however, its availability is significantly limited by the shortage of donor organs. As a result, only about 10% of eligible patients eventually undergo heart transplantation, with many dying while on the waiting list. In lower-middle-income countries, the challenges are further exacerbated, as financial constraints often make heart transplantation unfeasible, leaving many patients without access to this life-saving procedure. According to the literature, patients with severe LVD undergoing surgical interventions such as CABG demonstrate improved survival rates, enhanced functional status, and better control of ischemic symptoms postoperatively. Additionally, surgery has been associated with a reduced incidence of sudden cardiac deaths, particularly those related to arrhythmias, suggesting a protective effect against these adverse events [22-24].

In comparison to medical therapy alone, surgical interventions such as CABG have shown better outcomes, including improved survival, functional status, and control of ischemic symptoms. Evidence suggests that relying solely on medical therapies may result in poorer outcomes, highlighting the advantages of surgical management for this high-risk patient population. Despite the potential benefits, surgical coronary revascularization in patients with severe LVD carries a high risk of postoperative mortality, primarily due to perioperative low cardiac output syndrome. However, recent advancements in surgical techniques, enhanced myocardial protection strategies, and improved perioperative anesthetic care have led to better patient outcomes. These improvements have allowed a growing number of patients with severely impaired LV function to successfully undergo CABG.

Conventional on-pump CABG remains the standard technique but carries risks such as inflammatory responses, cardioplegia, aortic cross-clamping, hypothermia, and multi-organ dysfunction. Off-pump CABG has emerged as an effective alternative, reducing systemic ischemia and associated complications. While it offers advantages such as less myocardial manipulation, concerns about incomplete revascularization, graft patency, and long-term outcomes persist. In our study, the choice between on-pump and off-pump CABG was made individually based on hemodynamic stability, coronary disease severity, overall health, and the surgeon's preference. Various studies have shown that the use of on-pump versus off-pump CABG yields similar rates of morbidity and mortality, indicating that both approaches are relatively comparable in terms of patient outcomes [25,26]. Furthermore, the judicious use of inotropic support during and after surgery and complete myocardial revascularization likely supported these favorable outcomes.

Additionally, effective myocardial protection achieved through both antegrade and retrograde cardioplegia during isolated CABG and CABG with VSR repair proved to be key factors in improving short-term results in patients with poor LV function. These findings emphasize that comprehensive surgical strategies and careful intraoperative management are critical predictors of successful outcomes in this high-risk patient population.

This study has several limitations. Its retrospective design may introduce selection bias, and being a single-center study limits generalizability. While the sample size of 454 patients is substantial, it may still be inadequate for subgroup analyses. The study focused on short-term outcomes, with limited long-term follow-up data. Variations in surgical techniques and unmeasured confounders may have influenced results. These factors highlight the need for multicenter, prospective studies with longer follow-ups to confirm the findings. This study will be expanded to include a larger patient population and to evaluate long-term survival outcomes and changes during the extended postoperative period.

## Conclusions

CABG remains a vital surgical intervention for patients with severe LVD, offering favorable short-term outcomes despite the inherent risks. Key factors contributing to these results include comprehensive myocardial revascularization, effective use of internal mammary artery grafting, and advanced myocardial protection strategies. This study highlights the potential of CABG to improve survival and functional outcomes in this high-risk population. However, further multicenter research with larger cohorts and long-term follow-up is needed to confirm these findings and refine surgical approaches for optimizing outcomes in patients with complex CAD and LVD.
